# Changes in Functional Integration with the Non-Epileptic Temporal Lobe of Patients with Unilateral Mesiotemporal Epilepsy

**DOI:** 10.1371/journal.pone.0067053

**Published:** 2013-06-20

**Authors:** Nicola Trotta, Serge Goldman, Benjamin Legros, Kristof Baete, Koen Van Laere, Patrick Van Bogaert, Xavier De Tiège

**Affiliations:** 1 Laboratoire de Cartographie fonctionnelle du Cerveau, Hôpital Erasme, Université Libre de Bruxelles (ULB), Brussels, Belgium; 2 Department of Nuclear Medicine, Hôpital Erasme, Université Libre de Bruxelles (ULB), Brussels, Belgium; 3 Reference Centre for Refractory Epilepsy, Hôpital Erasme, Université Libre de Bruxelles (ULB), Brussels, Belgium; 4 Department of Nuclear Medicine, UZ Leuven, Leuven, Belgium; University of Manchester, United Kingdom

## Abstract

**Purpose:**

To investigate epilepsy-induced changes in effective connectivity between the non-epileptic amygdalo-hippocampal complex (AHC) and the rest of the brain in patients with unilateral mesiotemporal lobe epilepsy (MTLE) associated with hippocampal sclerosis (HS).

**Methods:**

Thirty-three patients with unilateral MTLE associated with HS (20 females, mean age: 36 years, 19 left HS) and 33 adult controls matched for age and gender underwent ^18^F-Fluorodeoxyglucose positron emission tomography (FDG-PET). Right-HS patients' FDG-PET data were flipped to obtain a left–epileptic–focus–lateralized group of patients. Voxels of interest (VOI) were selected within the cytoarchitectonic probabilistic maps of the non-epileptic AHC (probability level  = 100%, SPM8 Anatomy toolbox v1.7). Patients and controls were compared using VOI metabolic activity as covariate of interest to search for epilepsy-induced changes in the contribution of the non-epileptic AHC to the level of metabolic activity in other brain areas. Age, gender, duration of epilepsy, seizure type and frequency were used as covariates of no-interest for connectivity analyses.

**Key findings:**

Significant decrease in effective connectivity was found between the non-epileptic AHC and ventral prefrontal cortical areas bilaterally, as well as with the temporal pole and the posterior cingulate cortex contralateral to HS. Significant increase in connectivity was found between the non-epileptic AHC and midline structures, such as the anterior cingulate and dorsal medial prefrontal cortices, as well as the temporo-parietal junction bilaterally. Connectivity analyses also revealed a preserved positive connectivity between the non-epileptic and the epileptic AHC in the patients' group.

**Significance:**

This study evidences epilepsy-induced changes in connectivity between the non-epileptic AHC and some limbic and default mode network areas. These changes in connectivity probably account for emotional, cognitive and decision-making impairments frequently observed in MTLE patients. The preserved neurometabolic connectivity between the non-epileptic and the epileptic AHC in MTLE patients is pivotal to explain the epilepsy-induced changes found in this study.

## Introduction

The functional integration within and between neuronal networks is crucial for normal information processing and behaviors, but it may also ground the pathophysiological basis of long-range neuronal functional disturbances and functional networking reorganization associated with focal brain disorders [1].

Mesial temporal lobe epilepsy (MTLE) is an epileptic syndrome characterized by recurrent partial seizures arising form mesiotemporal structures [2]. Unilateral hippocampal sclerosis (HS) is the most frequent MTLE pathological substrate, representing the structural abnormality in 60% to 80% of patients [2]. MTLE is the most common type of pharmacoresistant partial epilepsy in adults, for which resective surgery represents a widely accepted and effective therapeutic option [3].

Positron emission tomography studies using ^18^F-Fluorodeoxyglucose (FDG-PET) performed interictally in patients with unilateral MTLE have demonstrated significant hypometabolism in the epileptic temporal regions in about 60% to 95% of the patients [4]–[7]. Significant metabolic changes have been concomitantly observed in surrounding and remote brain regions such as lateral temporal areas, prefrontal cortex and thalamus ipsilateral to HS [6]–[10]. These data indicate that, in MTLE, the spatial extent of interictal hypometabolic brain areas is more widespread than the actual epileptogenic zone associated with hippocampal neuronal loss. Animal studies indicate that the decreased glucose consumption observed in brain areas surrounding or remote from the epileptogenic zone is related to functional inhibition rather than epileptogenesis *per se* [11]. Such inhibition phenomena have been well documented in different animal models of focal epilepsy using various functional cerebral imaging methods such as autoradiography or optical imaging [11], [12]. Their occurrence in human epilepsy has been suspected in MTLE as well as in extra-temporal lobe epilepsy on the basis of data obtained with FDG-PET, electroencephalography (EEG) combined with functional magnetic resonance imaging (MRI) or single photon emission computed tomography [1], [9], [13], [14]. In particular, increasing evidence suggests that MTLE is associated with altered metabolism in the frontal lobe and some brain areas belonging to the default mode network (DMN), which might be related to specific epilepsy-induced changes in effective connectivity involving those structures [1], [9], [14]–[16]. From a clinical perspective, hypometabolic brain areas distant from the mesiotemporal regions might account for some of the cognitive impairments frequently observed in MTLE patients as suggested by the regression of distant hypometabolism [17]–[19] and the improvement of non-memory function impairments after surgical treatment of MTLE [20], [21].

Besides the occurrence of this neuronal inhibition, increasing evidence supports the existence of disease-induced plasticity mechanisms in the non-epileptic hemisphere and, more particularly, in the non-epileptic temporal lobe of patients with unilateral MTLE [6], [7], [22]–[25]. These plasticity phenomena have been associated with better performances in memory or speech functions [6], [22], [25]. Although not consistently observed in all studies, these data suggest that the occurrence of functional reorganization in the non-epileptic temporal lobe might represent a compensatory mechanism sustaining some key cognitive functions such as memory or speech.

Taken together, all these observations indicate that the consequences of unilateral MTLE are not confined to the epileptogenic zone but rather extend to distributed networks, possibly via disease-induced changes in functional integration and plasticity phenomena. The main purpose of this study was to better characterize in a large group of patients the remote consequences of unilateral MTLE by searching for disease-induced changes in functional integration in brain areas not primarily involved in the epileptic disorder. Based on the established neuroanatomy of the amygdalo-hippocampal complex (AHC) [26] and existing human experimental data suggesting disease-induced functional changes in the non-epileptic temporal lobe in MTLE [6], [7], [14]–[16],[22], we hypothesized the existence of significant changes in effective connectivity between the non-epileptic AHC and related limbic or DMN areas. The connectivity analyses were restricted to the non-epileptic AHC in order to avoid the effects of the gross anatomical changes associated with HS that complicate connectivity analyses from the epileptic AHC.

## Patients and Methods

### Ethics Statement

Patients' FDG-PET data were obtained in the context of a multidisciplinary presurgical evaluation for refractory focal epilepsy. The ULB-Hôpital Erasme Ethics Committee gave approval for conducting and using these FDG-PET data in this retrospective study. The ULB-Hôpital Erasme Ethics Committee waived the requirement for obtaining patients' or parents' (on the behalf of children participants) informed consent (oral and written) in the context of this retrospective study. The Institutional Ethics Committees gave approval for the FDG-PET investigations obtained in adult controls. Written informed consent was obtained from all control subjects.

### Patients & control subjects

Among the population of patients studied by FDG-PET between February 2000 and May 2009 at the PET/Biomedical Cyclotron Unit of the ULB-Hôpital Erasme, we retrospectively selected 33 patients (20 women and 13 men, aged 7 to 61 years, mean age 36.06 years) based on the following inclusion criteria: 1) refractory focal epilepsy included in the multidisciplinary presurgical evaluation program of the ULB-Hôpital Erasme, 2) five days of scalp video-EEG suggesting unilateral MTLE, 3) unilateral HS on structural cerebral MRI, and 4) absence of neurological condition other than epilepsy. All patients underwent neuropsychological evaluation and interictal FDG-PET. During the video-EEG monitoring sessions, we recorded an average of 3.75±2.9 seizures-per-patient. In two patients, no seizure was recorded. Based on the multidisciplinary presurgical evaluation, it was concluded that the epileptic focus was unilateral and located in the mesiotemporal region in all patients. Nineteen patients had a left MTLE and fourteen had a right MTLE. Duration of epilepsy ranged from 2 to 54 years with a mean duration of 25.15 years. Surgical procedures were performed in 28 patients (anterior temporal and amygdalo-hippocampal disconnection in 22, radiosurgery in 6). Outcome for seizures (follow-up ranging from 3 months up to 11 years; 1 patient not yet evaluated) showed 19 patients (68%) in Engel class I (17 IA and 2 IC), 3 in Engel class IIA, 5 in Engel class III (4 IIIA and 1 IIIB) [27].

A group of 33 healthy adult volunteers matched to obtain similar age mean and gender proportion with patients' group (20 women and 13 men, aged 20 to 50 years, mean age 36.03 years) was used as control population. FDG-PET data of 23 subjects were acquired at the PET/Biomedical Cyclotron Unit of the ULB-Hôpital Erasme. FDG-PET data of the other subjects were acquired at the Nuclear Medicine division of the UZ Leuven. All FDG-PET data were acquired on the same type of PET camera using the same data acquisition and reconstruction procedure.

### FDG-PET data acquisition

FDG-PET data were acquired using an ECAT 962 Exact HR+ camera (CTI-Siemens, Knoxville, TX), the characteristics of which have been previously described [28]. The patients' anti-epileptic treatment (Table 1) was not changed for the FDG-PET evaluation. Routine EEG monitoring was not performed during the scan. Patients were, however, asked to report any seizures experienced on the day of the PET scan, whether before or after the FDG injection, as well as the 3 days before [29]. Only one patient experienced a partial complex seizure 4 hours before the FDG-PET acquisition. The other patients did not report any clinical seizure during the 3 days before FDG-PET. All patients fasted for at least 4 hours, were awake, in eye-closed rest and placed in a quiet dark room. They received an intravenous bolus injection of 2 to 5 mCi (74 to 185 Mbq) of FDG 40 minutes before a 20-minute PET data acquisition in three-dimensional mode. One emission frame composed by 63 transaxial slices was obtained and realigned to the canthomeatal line. Images were corrected for photon attenuation thanks to a post-injection 10-minute transmission scan obtained with Ge-68 line sources. Each PET image was reconstructed using filtered back projection and displayed in a 128×128×63 voxel format, with a slice thickness of 2.4 mm and an in-plane resolution of 4.6 mm.

**Table 1:** Patients' anti-epileptic treatment.

Anti-epileptic drug	N° of patients
levetiracetam	20
carbamazepine	16
topiramate	10
lamotrigine	9
valproic acid	7
oxcarbazepine	6
phenytoin	4
phenobarbital	3
gabapentin	2
phenytoin+phenobarbital	1
benzodiazepin	1

### FDG-PET data analyses

FDG-PET data were analyzed using the voxel-based Statistical Parametric Mapping method (SPM8, http://www.fil.ion.ucl.ac.uk/spm/, Wellcome Department of Imaging Neuroscience, London, UK). The PET images were spatially normalized to the Montreal Neurologic Institute template (Montreal Neurologic Institute, Quebec, Canada). The scans were then smoothed using a 12-mm full-width at half-maximum isotropic kernel. Global activity normalization was performed by proportional scaling [7].

In order to identify the effective connectivity changes between the non-epileptic AHC and the rest of the brain that were common to patients with left and right-sided MTLE, we horizontally flipped the FDG-PET scans of patients with right MTLE to lateralize the epileptogenic zone on the same side in all patients [7]. Therefore, we obtained a group of patients with “left-sided” epileptogenic zone by selecting either the original scans (19 patients with left MTLE) or the mirror scans (14 patients with right MTLE whose scans had been horizontally flipped). To avoid any confounding effect of this flipping procedure on the effective connectivity comparisons between patients and controls, the FDG-PET scans of 14 control subjects matched for the sex of the right MTLE patients were horizontally flipped.

For effective connectivity analyses, voxels of interest (VOIs) were selected within the non-epileptic AHC using the cytoarchitectonic probability maps of the SPM Anatomy Toolbox 1.7b (Institut für Neurowissenschaften und Medizin (INM), Jülich Forschungszentrum, http://www2.fz-juelich.de/inm/inm-1/spm_anatomy_toolbox). Based on MTLE pathophysiology, 2×2×2 mm^3^ VOIs were arbitrarily selected in the 100% probability maps [30] of the right non-epileptic cornu ammonis (three voxels along the antero-posterior axis: (32-12-26), (32-18-20) and (28-38-4)), the entorhinal cortex (three voxels along the antero-posterior axis: (22-2-40), (24-6-38) and (24-20-30) and the amygdala (centromedial nucleus: (24-10-8), laterobasal nucleus: (24-6-26) and superficial nucleus (18-4-18)).

Pathophysiologic interactions (PathoPI) were used for effective connectivity analyses in order to search for disease-related changes in the contribution of a brain area to the level of metabolic activity in another brain area in patients compared to controls [13], [31]. As such, this analysis is an application of the previously developed psychophysiological interaction analysis to a pathologic condition [32]. PathoPI analyses were conducted based on the *a priori* hypothesis of an altered effective connectivity between the non-epileptic AHC and related limbic (medial prefrontal cortex, anterior cingulate cortex, orbitofrontal cortex, thalamus, hypothalamus) [33], [34] and DMN areas [35]. In practice, for each VOI, the VOI metabolic activity was introduced in the design matrix as a covariate of interest centred around condition mean and interacting with each condition. Separate *t*-contrast analyses then searched, throughout the brain, for regions showing significantly lower or higher activity modulation with the considered VOI in the patients' group than in controls. To assess a potential effect of age, gender, main seizure type (simple partial, simple complex or secondary generalized seizures), seizure frequency and duration of epilepsy on the connectivity changes, PathoPI analyses were also performed with each of these variables used as covariates of no-interest.

Regression plots of the brain areas showing significant effective connectivity changes with the VOIs were obtained in Matlab 7.6 R2008a (Mathworks Inc., Sherborn, MA, USA). After a linear interpolation of the regression plots, the associated slope coefficients and *p*-values were calculated using the *corrcoef* function of the Matlab statistical toolbox version 6.2 (Matlab 7.6 R2008a, Mathworks Inc.).

Finally, in order to better understand the pathophysiological mechanisms involved in the epilepsy-induced changes in connectivity, subtractive SPM analyses were conducted at the group level to compare the PET data of the patients taken as a group with those of the control group [7], [13]. For these analyses, the design matrix separately included the 33 left-lateralized patients' FDG-PET scans (19 original and 14 flipped) taken as a group and the FDG-PET scans of the control group (19 original and 14 flipped). Separate *t*-contrast analyses identified brain regions where glucose metabolism was significantly lower or higher in the group of patients than in the control population.

All results of SPM analyses were considered significant at *p*<0.05 corrected for multiple comparisons over the entire brain volume (Family Wise Error, FWE). For effective connectivity analyses, we also considered significant at a small volume-corrected (SVC) *p*<0.05 (10-mm radius spherical volume of interest) voxels located in limbic [34], [36] and DMN [35] areas if they displayed significant correlation coefficients (*p*<0.05) with the considered VOI in the control population.

## Results

### Decrease in effective connectivity

**Figure 1:**
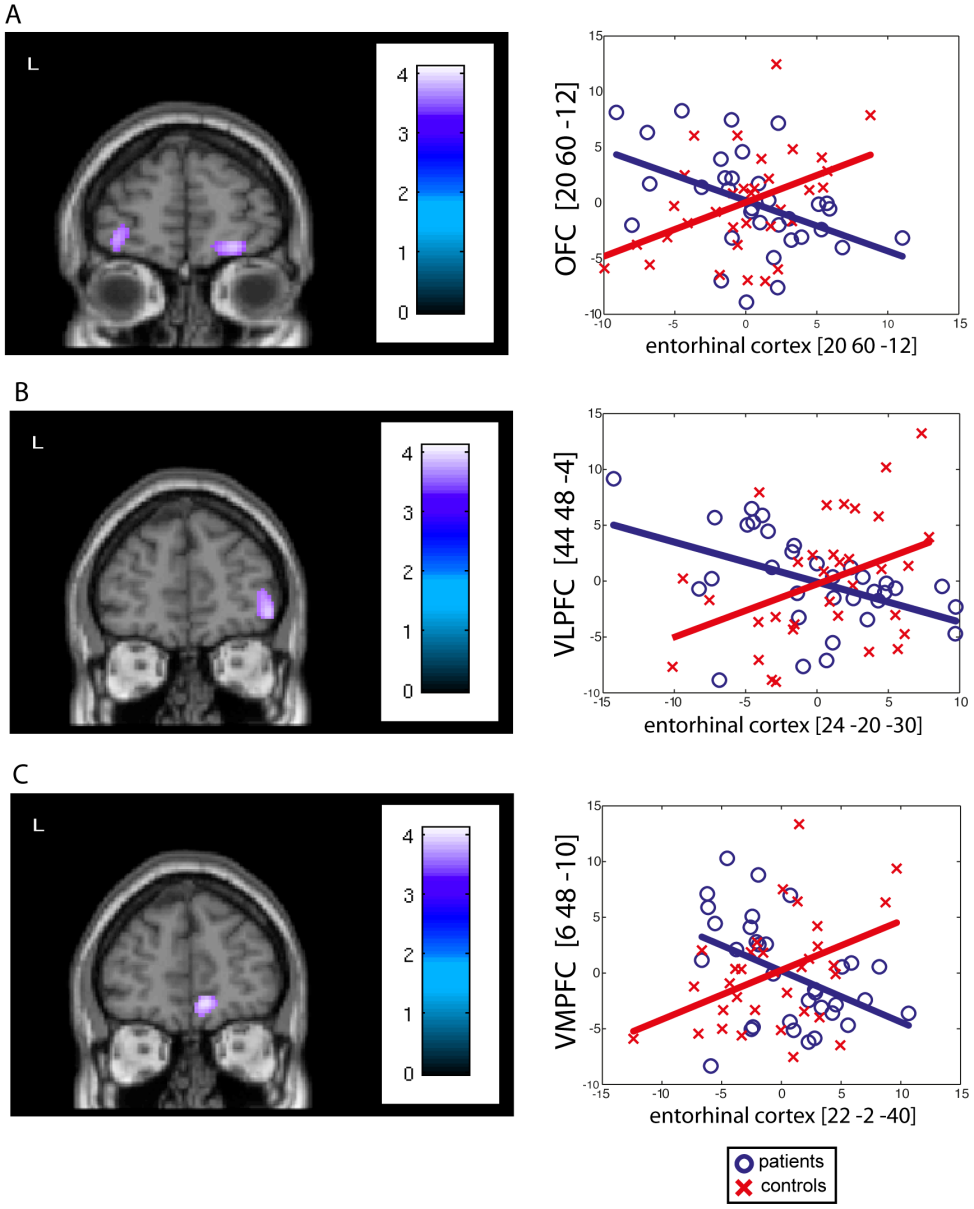
Significant decreases in effective connectivity. Significant decreases in connectivity were mainly found between the non-epileptic AHC and (A) the orbitofrontal cortex ([20 60 −12], Pearson's correlation: r = −0.45, p 0.002), (B) the ventral lateral prefrontal cortex ([44 48 −4], Pearson's correlation: r = −0.46, p = 0.007) and (C) the ventral medial prefrontal cortex ([6 48 −10], Pearson's correlation: r = −0.44, p = 0.005). Regression plots (right side of the figure) of the metabolic activity in the non-epileptic AHC and fronto-limbic areas were obtained in control subjects (red crosses) and in the group of patients (blue circles). These regression plots illustrate the epilepsy-induced changes in the contribution of the non-epileptic AHC to the level of metabolic activity in fronto-limbic areas.

Table 2 and Figure 1 detail the significant decrease in effective connectivity observed between each VOI and the rest of the brain in patients compared to controls.

**Table 2:** Significant decreases in effective connectivity.

Seed Voxel				Voxels showing significant PathoPI				*p* values^a^		*r*
Region	MNI			Region	MNI atlas					
	x	y	z		x	y	z			
CA anterior	32	−12	−26	L T pole	−30	12	−38		0.017	−0,31
CA middle	32	−18	−20	L T pole	−30	14	−36		0.005	−0,38
CA posterior	28	−38	−4	−						
EC anterior	22	−2	−40	R OFC	20	62	−12		0.003	−0,46
				R VMPFC	6	48	−10		0.005	−0,44
EC middle	24	−6	−38	R OFC	20	60	−12		0.002	−0,45
				L OFC	−36	56	−6		0.010	−0,39
				R OFC	42	50	−2		0.015	−0,46
EC posterior	24	−20	−30	R VLPFC	44	48	−4		0.007	−0,46
				L VMPFC	−10	66	24		0.010	−0,35
				L OFC	−38	56	−12		0.013	−0,39
Amyg CM	24	−10	−8	L PCC	−14	−54	20		0.003	−0,39
Amyg LB		−6	−26	L T pole	−30	12	−38		0.007	−0,31
	24			R OFC	10	20	−26		0.018	−0,27
				L OFC	−32	28	−20		0.019	−0,25
				R OFC	18	40	−20		0.022	−0,26
Amyg SF	18	−4	−18	-						

R: right; L: left; CA: cornu ammonis; EC: enthorinal cortex; Amyg: amygdala; CM: centromedial nucleus; LB: laterobasal nucleus; SF superficial nucleus; T pole: temporal pole; OFC: orbitofrontal cortex; VMPFC: ventromedial prefrontal cortex; VLPFC: ventrolateral prefrontal cortex; PCC: posterior cingulate cortex. ^a^p^corrSVC^. *r*: Pearson's correlation coefficient between seed voxel and identified area.

The most consistent connectivity changes were significant decreases in connectivity between the non-epileptic AHC and ventral prefrontal cortical areas such as the orbitofrontal cortex (OFC) bilaterally (enthorinal cortex, amygdala), the ventral medial prefrontal cortex (VMPFC) bilaterally (enthorinal cortex) and the ventral lateral prefrontal cortex (VLPFC, enthorinal cortex) in the non-epileptic hemisphere. Altered connectivity was also found with the temporal pole (cornu ammonis, amygdala) and the posterior cingulate cortex (PCC, amygdala) in the epileptic hemisphere.

### Increase in effective connectivity

**Figure 2:**
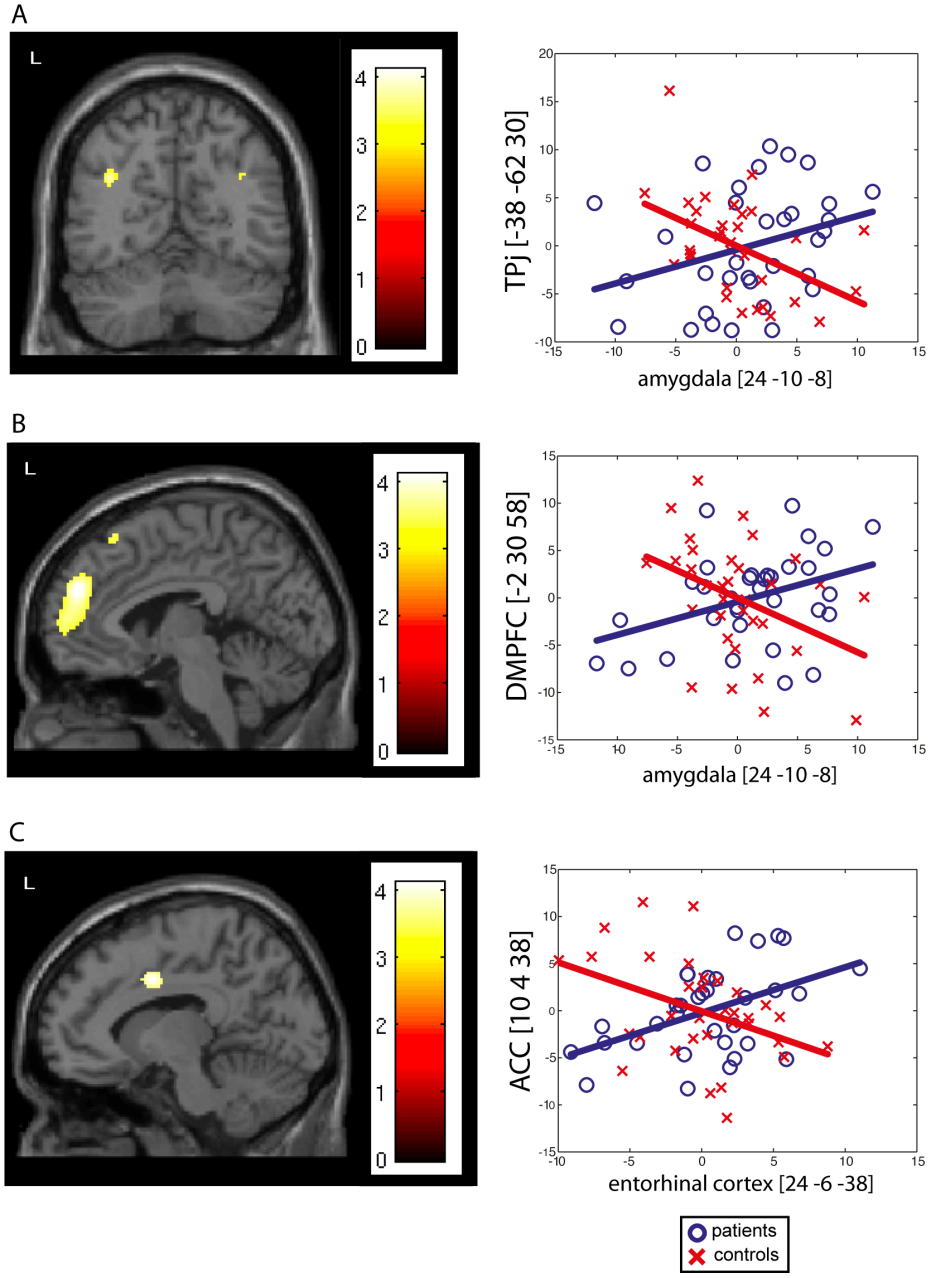
Significant increases in effective connectivity. Significant increases in connectivity were mainly found between the non-epileptic AHC and (A) the anterior cingulate cortex ([10 4 38], Pearson's correlation: r = 0.47, p = 0.003), (B) the dorsal medial prefrontal cortex ([−2 30 58], Pearson's correlation: r = 0.38, p = 0.004), and (C) the temporo-parietal junction ([−38 −62 30], Pearson's correlation: r = 0.31, p = 0.004). Regression plots (right side of the figure) of the metabolic activity in the non-epileptic AHC and these DMN areas were obtained in control subjects (red crosses) and in the group of patients (blue circles). These regression plots illustrate the epilepsy-induced changes in the contribution of the non-epileptic AHC to the level of metabolic activity in these DMN areas.

Table 3 and Figure 2 detail the significant increase in effective connectivity observed between VOIs and the rest of the brain in patients compared to controls.

**Table 3:** Significant increases in effective connectivity.

Seed Voxel				Voxels showing significant PathoPI				*p* values^a^	*r*	
Region	Talairach			Region	Talairach					
	x	y	z		x	y	z			
CA anterior	32	−12	−26	-						
CA middle	32	−18	−20	-						
CA posterior	28	−38	−4	L ACC	−12	28	34	0.009		0,38
EC anterior	22	−2	−40	R ACC	10	4	38	0.014		0,40
EC middle	24	−6	−38	R ACC	10	4	38	0.003		0,47
EC posterior	24	−20	−30	-						
Amyg CM	24	−10	−8	R DMPFC L DMPFC L TP junction R TP junction	4 −2 −38 36	52 30 −62 −64	28 58 30 32	0.001 0.004 0.004 0.019		0,360,380,310,28
Amyg LB	24	−6	−26	−						
Amyg SF	18	−4	−18	-						

R: right; L: left; CA: cornu ammonis; EC: enthorinal cortex; Amyg: amygdala; CM: centromedial nucleus; LB: laterobasal nucleus; SF superficial nucleus; ACC: anterior cingulate cortex; DMPFC: dorsomedial prefrontal cortex; TP: temporo-parietal. ^a^p^corrSVC^. *r*: Pearson's correlation coefficient between seed voxel and identified area.

In patients compared to controls, PathoPI analyses revealed significant increase in connectivity between the non-epileptic AHC and the anterior cingulate cortex (ACC; cornu ammonis, enthorinal cortex), the dorsal medial prefrontal cortex (DMPFC; amygdala), and the temporo-parietal junction bilaterally (amygdala).

### Effective connectivity between the non-epileptic and the epileptic AHC

**Figure 3:**
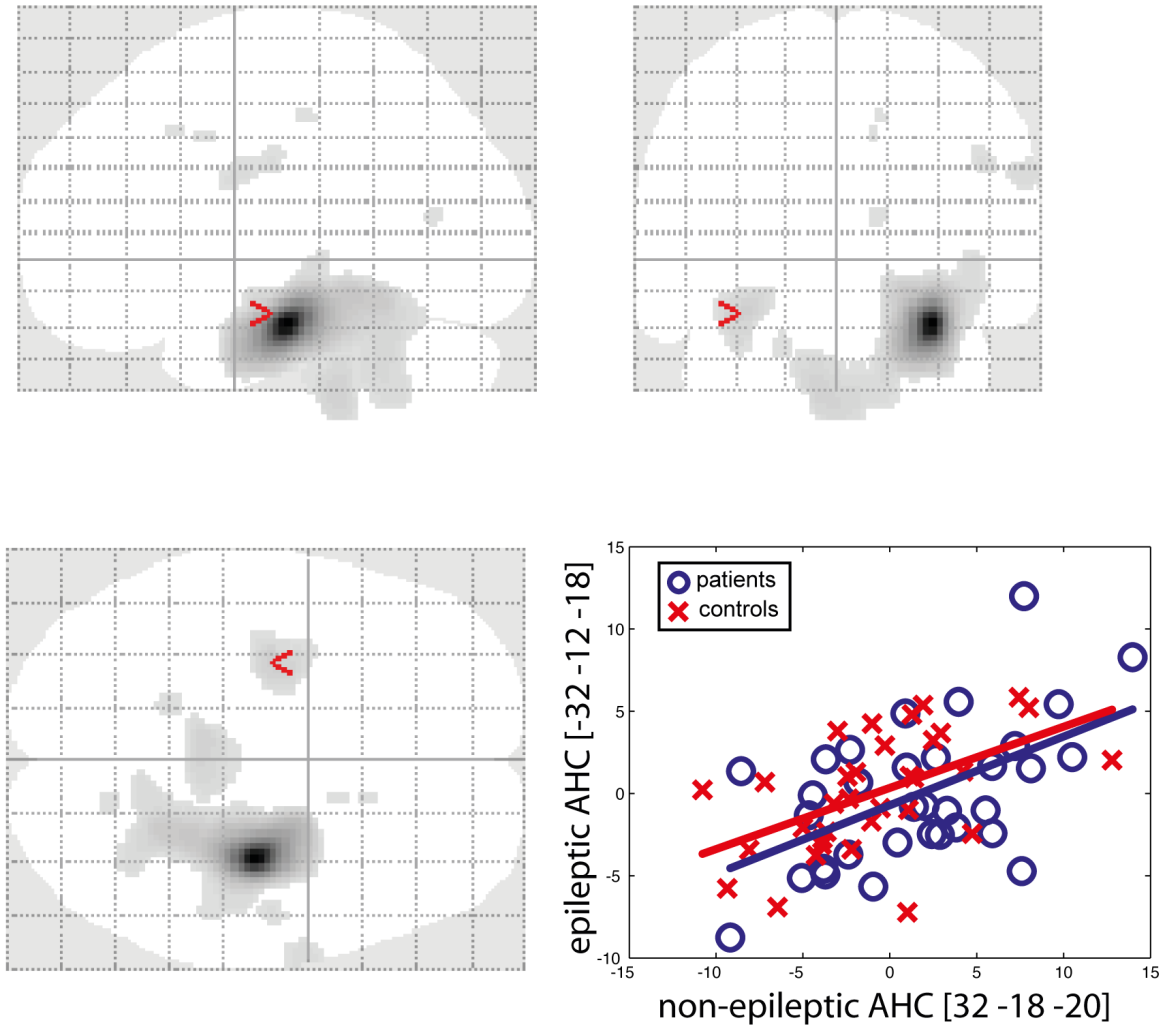
Effective connectivity between the non-epileptic and the epileptic AHC. The glass brain images (top, bottom left) show the brain areas presenting significant positive correlation with the non-epileptic AHC in the patients' group. These analyses revealed preserved positive connectivity between the non-epileptic and epileptic AHC in the patient's group. Regression plots (bottom right) of the metabolic activity in the non-epileptic and epileptic AHC were obtained in control subjects (red crosses) and in the group of patients (blue circles). The regression plot illustrates the preserved contribution of the non-epileptic AHC to the level of the metabolic activity in the epileptic AHC ([−32 −12 −18], Pearson's correlation for the patients' population: r = 0.54, p = 0.001).

Figure 3 illustrates the results of connectivity analyses between the non-epileptic AHC and the epileptic AHC.

As PathoPI analyses did not show significant changes in effective connectivity between the right non-epileptic and the left epileptic AHC, separate *t*-contrast analyses searched, throughout the brain, for regions showing significant positive correlation with the considered VOI in the patients' group. These analyses did not disclose significant changes in connectivity between the non-epileptic and epileptic AHC in the patient's group.

### Effect of age, gender, seizure type and duration of epilepsy on connectivity changes

Introducing age, gender, main seizure type (20 patients with complex partial seizures and 13 with secondary generalization), seizure frequency and duration of disease as covariates of no-interest in PathoPI analyses did not modify the results.

### Subtractive group-level analyses

Significant hypometabolic areas were found in the mesial and lateral temporal lobe, the VLPFC and the inferior parietal lobule ipsilateral to the epileptogenic zone as well as in the ACC bilaterally. In addition, significant hypermetabolic areas were also found in the inferior temporal gyrus contralateral to the epileptic hemisphere.

## Discussion

The voxel-based analyses of changes in effective connectivity between the non-epileptic AHC and the rest of the brain in patients with unilateral MTLE associated with unilateral HS disclosed the following findings: (1) preserved connectivity between the non-epileptic and the epileptic AHC, (2) significant decrease in effective connectivity between the non-epileptic AHC and the ventral prefrontal cortex, as well as with the temporal pole and the PCC contralateral to HS, and (3) significant increase in connectivity between the non-epileptic AHC and midline structures such as the DMPFC and ACC, as well as the temporo-parietal junction bilaterally.

In accordance with previous FDG-PET studies performed in patients with unilateral MTLE, we found significant relative decrease in metabolism in the epileptic temporal lobe, in extratemporal brain areas ipsilateral to the epileptic focus such as the VLPFC and the inferior parietal lobule as well as in the ACC bilaterally. We also found significant relative increase in metabolism in the temporal lobe contralateral to the epileptic focus potentially attributable, as previously proposed, to epilepsy-induced functional reorganization or plasticity phenomena [6], [7], [37]. Being in line with a large literature on brain glucose metabolic changes in MTLE, these results substantiate that the group of patients studied is highly representative of the population of patients with unilateral MTLE associated with HS.

### Effective connectivity between the non-epileptic and the epileptic AHC

The PathoPI analyses did not show any significant alterations in neurometabolic connectivity between the non-epileptic and the epileptic AHC. This finding strongly suggests that the mesiotemporal structures remain tightly connected despite the unilateral HS and the associated recurrent seizures. This preserved connectivity is pivotal to explain the functional integration changes found between the non-epileptic AHC and some specific brain areas. Indeed, by allowing the propagation of epileptiform activity towards the non-epileptic AHC, this preserved connectivity might lead to repetitive alterations in neuronal activity at the level of the non-epileptic AHC [38], potentially inducing chronic changes in effective connectivity stemming from this structure. This finding therefore brings further support to the hypothesis that, in MTLE, it is the seizure propagation pathways that determine the distribution of functional and structural damages [39] rather than the loss of hippocampal connections [40]. Nevertheless, previous resting state functional MRI (rsfMRI) studies that have investigated the cross-AHC connectivity in patients with unilateral MTLE have found conflicting results [41]–[44]. This discrepancy might be related to differences in patients' populations and methods, to variable effects of the disease on neurovascular coupling, to variations in the amount of interictal epileptic discharges [39] during rsfMRI data acquisition or to variations in epilepsy duration. The FDG–PET method used in this study, which investigates the neurometabolic coupling and which is characterized by a much lower temporal resolution than fMRI-with less immediate influence of intermittent interictal epileptiform discharges [45] —, probably overcomes some of the methodological limitations associated with rsfMRI in MTLE. Contrary to the results from a recent rsfMRI study performed in patients with unilateral MTLE [42], we did not find any effect of epilepsy duration on cross-AHC connectivity. So, the duration of epilepsy does not seem to influence the AHC neurometabolic connectivity in patients with unilateral MTLE.

### Decrease in effective connectivity

The PathoPI analyses mainly revealed a significant decrease in effective connectivity between the non-epileptic AHC and ventral prefrontal structures such as the OFC, the VMPFC and the VLPFC. These brain areas are parts of the neural networks involved in emotion processing and regulation, decision-making or reward processing [36], [46], [47]. A recent rsfMRI study performed in patients with unilateral MTLE also revealed significant decrease in connectivity between the non-epileptic hippocampus and ventral mesial prefrontal areas [44], strengthening our finding. In addition, we found altered connectivity with the temporal pole and the PCC that are also parts of the neural network subserving cognitive, emotional and decision-making processes [48]–[50]. Interestingly, several studies have demonstrated the existence of significant deficits in cognition (including social cognition) and emotional processes in patients with MTLE compared to patients with extra-temporal epilepsy or healthy control subjects [51]–[54]. In addition, impairment in decision-making abilities, more particularly in the context of decisions under ambiguity, has also been reported in MTLE patients [55]–[57]. The epilepsy-induced changes in functional integration found in this study might therefore represent a neuronal correlate of these cognitive, emotional and decision-making impairments frequently found in patients with MTLE. Moreover, an FDG-PET study showed that MTLE patients with a pre-operative history of depression were characterized by significant hypometabolism in the orbitofrontal cortex compared with those who did not [58]. Therefore, alteration in the functional integration between the non-epileptic AHC and the orbitofrontal cortex could also potentially participate to the development of some psychiatric comorbidities of MTLE, such as depression or negative symptoms that are often associated with this epileptic disorder [59]. Further studies investigating the relationship between the connectivity changes found in the study and the comorbidities associated with unilateral MTLE should address this question by testing correlations between neuronal activity and relevant cognitive or behavioral parameters.

Regarding the pathophysiological mechanisms that would link the changes in effective connectivity found in this study with the patients' epileptic disorder, it is noteworthy that most of the brain areas showing significant decrease in connectivity with the non-epileptic AHC did not show any substantial change in relative glucose metabolism compared to the control group. This finding suggests that the decrease in connectivity observed with the non-epileptic AHC is directly related to an epilepsy-induced alteration of the non-epileptic AHC function with subsequent decrease in normal functional integration with connected brain areas, rather than to a direct influence of the epileptic AHC on brain areas connected with the non-epileptic AHC via, for example, a mechanism of remote inhibition.

### Increase in effective connectivity

The PathoPI analyses also revealed a significant increase in effective connectivity between the non-epileptic AHC and dorsal medial prefrontal areas as well as the temporo-parietal junction bilaterally. These regions are known to be part of the DMN, a network subserving an organized mode of brain function being present as a baseline or default state and attenuated during specific goal-directed tasks [35].

The epilepsy-induced increase in connectivity between the non-epileptic AHC and DMN nodes is quite challenging to explain. It might be the reflection of the reorganized contribution of the non-epileptic AHC to the multiple networks in which it normally participates. Such reorganization might result from compensatory mechanisms in the presence of unilateral HS, as proposed to explain the enhanced connectivity between the right non-epileptic medial temporal lobe and ipsilateral frontal and temporal areas during a working memory task performed by patients with MTLE from a left-sided HS [60]. Considering the complex and dynamical organization of brain functional networks [61], the increased connectivity of the non-epileptic AHC with the DMN might also arise from the imbalance produced by the decreased connectivity of AHC with areas involved in cognitive and emotional networks.

### Limitation of the study

We investigated the effective connectivity changes between the non-epileptic AHC and the rest of the brain that were common to patients with left and right-sided MTLE. For that purpose, we horizontally flipped the FDG-PET scans of patients with right MTLE to lateralize the epileptogenic zone on the same side (left side) in all patients. Still, functional and anatomical connectivity patterns are not integrally symmetrical in human brain hemispheres (for a review, see [62]), so the use of flipped scans in the patients' and control groups actually restricts potential findings to connectivity changes involving the symmetrical components. It precludes assessing epilepsy-induced changes in connectivity that are specific to patients with right or left MTLE. Such a specific analysis was not performed in this study due to the low sample of right and left MTLE patients respectively. Nevertheless, by demonstrating functional integration changes shared by all patients with unilateral MTLE associated with HS, this study sets the pathophysiological ground of the cognitive and behavioral impairments associated with MTLE regardless of HS location.

Given the retrospective nature of this study, patients' selection occurred over a large period of time (from 2000 to 2009). Therefore, the patients did not undergo uniform neuropsychological investigations and it was therefore difficult to correlate our FDG-PET findings with standardized neuropsychological data. Furthermore, no MRI-based atrophy correction of the FDG-PET data was performed in this study, due to the lack of uniform MRI data acquisitions across patients. These issues should be addressed in future prospective FDG-PET studies.

### Conclusions

This study discloses preserved neurometabolic connectivity between the non-epileptic and the epileptic AHC, significant decrease in effective connectivity between the non-epileptic AHC and limbic areas, as well as significant increase in connectivity with DMN regions. These epilepsy-induced changes in functional integration might represent a neuronal correlate of the cognitive, emotional and decision-making impairments frequently found in patients with unilateral MTLE.
