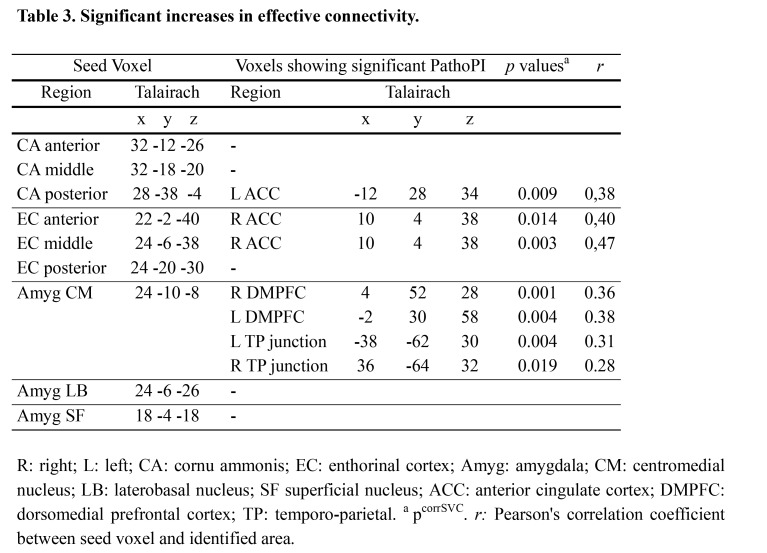# Correction: Changes in Functional Integration with the Non-Epileptic Temporal Lobe of Patients with Unilateral Mesiotemporal Epilepsy

**DOI:** 10.1371/annotation/4cc627a9-3fbe-440d-af8d-d2b642e1c188

**Published:** 2014-01-06

**Authors:** Nicola Trotta, Serge Goldman, Benjamin Legros, Kristof Baete, Koen Van Laere, Patrick Van Bogaert, Xavier De Tiège

A formatting error was introduced in Table 3 that may affect the readability of the table. Please see the corrected Table 3 here: 

**Figure:**